# Draft Genome Sequence of *Methylocaldum* sp. SAD2, a Methanotrophic Strain That Can Convert Raw Biogas to Methanol in the Presence of Hydrogen Sulfide

**DOI:** 10.1128/genomeA.00716-17

**Published:** 2017-08-10

**Authors:** Xiangdong Wei, Xumen Ge, Yebo Li, Zhongtang Yu

**Affiliations:** a Department of Animal Sciences, The Ohio State University, Columbus, Ohio, USA; b Department of Food, Agricultural and Biological Engineering, The Ohio State University, Columbus, Ohio, USA; c Department of Environmental Sciences, College of Resource & Environment, Hunan Agricultural University, Hunan, China

## Abstract

The draft genome sequence of *Methylocaldum* sp. SAD2, a methanotrophic strain isolated from a hydrogen sulfide-rich anaerobic digester, is reported here. Strain SAD2 possesses genes for methane oxidation in the presence of H_2_S.

## GENOME ANNOUNCEMENT

Hydrogen sulfide (H_2_S) is present in all biogas, which dramatically decreases the methane monooxygenase (MMO) activity and methane oxidation rate, inhibiting direct biological conversion of biogas by utilization of methanotrophs to produce liquid fuel (1, 2). Therefore, the need to isolate methanotrophic strains that can tolerate H_2_S is urgent. We recently isolated a methanotrophic strain, *Methylocaldum* sp. SAD2, from an H_2_S-rich anaerobic digester (3). Strain SAD2 is closely related to *Methylocaldum szegediense* and *Methylocaldum* sp. strain 14B (4), and it can directly convert biogas to methanol in the presence of H_2_S at concentrations up to 1,000 ppm (3). Here, we present the draft genome sequence of *Methylocaldum* sp. SAD2.

Cultivation, DNA extraction, and sequencing were done essentially as described previously (4). Briefly, *Methylocaldum* sp. SAD2 was grown in a nitrate mineral salts medium with a gas mixture containing 20% (vol/vol) CH_4_ and 80% (vol/vol) air (3). Genomic DNA was isolated using the cetyltrimethylammonium bromide (CTAB) method (5) and sequenced (2 × 300 bp) using an Illumina MiSeq system. Quality filtering using Trimmomatic version 0.35 (6) resulted in 2,179,853 paired-end reads. The filtered high-quality reads were *de novo* assembled into contigs using Newbler 2.9 (7). The assembly resulted in 96 contigs (*N*_50_, 132,078 bp; largest contig, 543,518 bp), with an average coverage of 110×.

The estimated draft genome size for *Methylocaldum* sp. SAD2 is 5,912,804 bp, with a G+C content of 58.18%. Genes were predicted from the assembled contigs using Glimmer 3.02 (8–10). Open reading frames (ORFs) were predicted using Prodigal (11). The predicted bacterial protein-coding sequences were searched using BLASTp against the Clusters of Orthologous Groups (COG) and GenBank (12) databases. Detection of tRNAs, rRNAs, and small RNA (sRNA) genes was performed using tRNAscan-SE 1.23 (13), RNAmmer 1.2 (14), and Rfam 10.1 (15), respectively. Tandem repeats were identified using Tandem Repeats Finder 4.04 (16). Minisatellite DNA and microsatellite DNA were predicted based on the number and length of repeat units. The draft genome contained one complete rRNA operon, 45 tRNA genes, 2 sRNA genes, 97 minisatellite DNAs, 10 microsatellite DNAs, 142 tandem repeat sequences, 4,834 genes with predicted functions, and 669 genes coding for hypothetical proteins. MegaBLAST searches (17) of the SAD2 concatenated genome against the NCBI reference genome database (http://www.ncbi.nlm.nih.gov/genome) revealed that the closest relative genome was *Methylocaldum szegediense* O-12 (GenBank accession no. NZ_ATXX00000000), with 89.44% sequence coverage and 94.83% sequence identity, and *Methylocaldum* sp. 14B (accession no. NZ_MSCV00000000), with 96.94% sequence coverage and 98.84% sequence identity.

Key genes associated with the pathways of methane oxidation, sulfur metabolism, relay system, and one-carbon assimilation (the serine cycle and ribulose-bisphosphate cycle) were identiﬁed, in agreement with the characterization data (3). Genes encoding enzymes of particulate methane monooxygenase, sulfide dehydrogenase, sulfurtransferase, sulfite oxidase and reductase, and sulfur transferase were also detected.

### Accession number(s).

This whole-genome shotgun project has been deposited at GenBank under the accession number MUGL00000000. The version described in this paper is the ﬁrst version, MUGL01000000.
